# Fabrication and characterization of Si_1−_*_x_*Ge*_x_* nanocrystals in as-grown and annealed structures: a comparative study

**DOI:** 10.3762/bjnano.10.182

**Published:** 2019-09-17

**Authors:** Muhammad Taha Sultan, Adrian Valentin Maraloiu, Ionel Stavarache, Jón Tómas Gudmundsson, Andrei Manolescu, Valentin Serban Teodorescu, Magdalena Lidia Ciurea, Halldór Gudfinnur Svavarsson

**Affiliations:** 1 Reykjavik University, School of Science and Engineering, IS-101 Reykjavik, Iceland; 2 National Institute of Materials Physics, 077125 Magurele, Romania; 3 Science Institute, University of Iceland, Dunhaga 3, IS-107 Reykjavik, Iceland; 4 Department of Space and Plasma Physics, School of Electrical Engineering and Computer Science, KTH Royal Institute of Technology, SE-100 44, Stockholm, Sweden; 5 Academy of Romanian Scientists, 050094 Bucharest, Romania

**Keywords:** grazing incidence XRD (GIXRD), high-power impulse magnetron sputtering (HiPIMS), HRTEM, magnetron sputtering, photocurrent spectra, SiGe nanocrystals in SiO_2_/SiGe/SiO_2_ multilayers, STEM-HAADF, TEM

## Abstract

Multilayer structures comprising of SiO_2_/SiGe/SiO_2_ and containing SiGe nanoparticles were obtained by depositing SiO_2_ layers using reactive direct current magnetron sputtering (dcMS), whereas, Si and Ge were co-sputtered using dcMS and high-power impulse magnetron sputtering (HiPIMS). The as-grown structures subsequently underwent rapid thermal annealing (550–900 °C for 1 min) in N_2_ ambient atmosphere. The structures were investigated using X-ray diffraction, high-resolution transmission electron microscopy together with spectral photocurrent measurements, to explore structural changes and corresponding properties. It is observed that the employment of HiPIMS facilitates the formation of SiGe nanoparticles (2.1 ± 0.8 nm) in the as-grown structure, and that presence of such nanoparticles acts as a seed for heterogeneous nucleation, which upon annealing results in the periodically arranged columnar self-assembly of SiGe core–shell nanocrystals. An increase in photocurrent intensity by more than an order of magnitude was achieved by annealing. Furthermore, a detailed discussion is provided on strain development within the structures, the consequential interface characteristics and its effect on the photocurrent spectra.

## Introduction

Currently, there is considerable interest in the growth of self-assembled quantum dots their application in optoelectronics and nanosized structures. For instance, semiconducting Si, Ge and SiGe nanocrystals (NCs/NPs) embedded in a dielectric oxide matrix have been found to exhibit strong quantum confinement. These NCs present unique and interesting size-dependent physical properties for a wide range of application including lighting, non-volatile memories, and electronic and photovoltaic applications [1–3]. SiGe nanostructures exhibit a stronger quantum confinement effect than Si NCs [4] and have the advantage of a bandgap fine-tuning by varying the Ge atomic fraction [5–6]. These properties are useful for optoelectronic devices working in the visible to far-infrared region [4,7].

Issues commonly observed with the fabrication of such structures include inhomogeneity at the matrix/nanoparticle (NCs/NPs) interfaces. Several studies have been devoted to the morphology of the interface between oxide matrices and NCs [8–10]. The interface of these structures has been a matter of concern in studying optical response as it may give rise to dangling bonds acting as electrically active interface traps (known as P_b_-type defects). These interface traps produce scattering centers that can affect the mobility of charge carriers, thus altering the transport properties [11]. Moreover, sharp interfaces with an abrupt change in the dielectric constant or thermal expansion coefficients give rise to surface polarization effects due to local fields, which play a crucial role in systems characterized by strong charge inhomogeneity. Further, the development of strain in the structure influences the size and shape of the NCs, thus resulting in a change of the bandgap energy.

A common method to obtain NCs embedded in an oxide matrix is by thermal annealing of multilayer structures. Several oxide matrices have been studied already [12–18], of which SiO_2_ is the most extensively studied as it remains amorphous up to high temperatures and due to its compatibility with Si-based technology [19–21]. Various fabrication methods have been utilized to fabricate structures with SiGe NCs embedded in an oxide matrix [13,17,22–23]. Magnetron sputtering is one of the most versatile methods and it allows for a good control over the NCs formation [24] by a addition of rapid thermal annealing. A rather recent variation of the magnetron sputtering technique, the so-called high-power impulse magnetron sputtering (HiPIMS), provides an alternative approach. It is an ionized physical vapor deposition method and has shown great promise in thin-film processing [25–26]. During HiPIMS the target is pulsed with short unipolar voltage pulses at low frequency and short duty cycle, achieving high discharge current densities leading to a high ionization fraction of the sputtered material [27–28]. This approach gives denser films [29] of higher crystallinity [30] than conventional direct current magnetron sputtering (dcMS) deposition technique.

Thermal treatment, being one of the most common methods to obtain NCs embedded in an oxide matrix, improves the efficiency and stability of the devices by altering the size of the embedded NCs [31–32]. In the present study, a short (1 min) rapid thermal annealing is carried out over earlier investigated structures [22], where the use of HiPIMS to obtain Si_1−_*_x_*Ge*_x_* NCs in as-grown samples is demonstrated. Upon rapid thermal annealing, periodically arranged columnar self-assembled SiGe NCs are obtained. The NCs are characterized using grazing incidence X-ray diffraction (GIXRD) and high-resolution transmission electron microscopy (HRTEM). Strain relaxation and its effect on the formation of NCs and the resulting interface integrity was studied and compared with structures having a thicker (ca. 200 nm) SiGe layer [23], deposited by radio-frequency magnetron sputtering (rfMS). In another previous study [22] we demonstrated NCs in as-grown structures with broader spectral response and improved efficiency after exposure to hydrogen plasma. The effect of annealing of such structures is yet to be explored, in order to preserve the functionality of devices containing such structures [32]. A comparison is made to present the effect of SiGe thickness on strain accumulation in NCs and demonstrate the effectiveness of mild thermal exposure, applicable to structures prone to decomposition at elevated temperatures.

## Results and Discussion

The multilayer structures (MLs) deposited in this study are similar to structures studied in our recent work [22] regarding stacking order (i.e., SiO_2_/SiGe/SiO_2_) and individual layer thicknesses. The difference in the fabrication is that during co-sputtering of the SiGe layer, we apply a lower cathode voltage for the Ge deposition, i.e., 445 V instead of 470 V, at a repetition frequency of 300 Hz, with an average power of 103 W. For Si (co-deposited via dcMS) the power is kept constant at 180 W.

### Structural analysis

**Figure 1:**
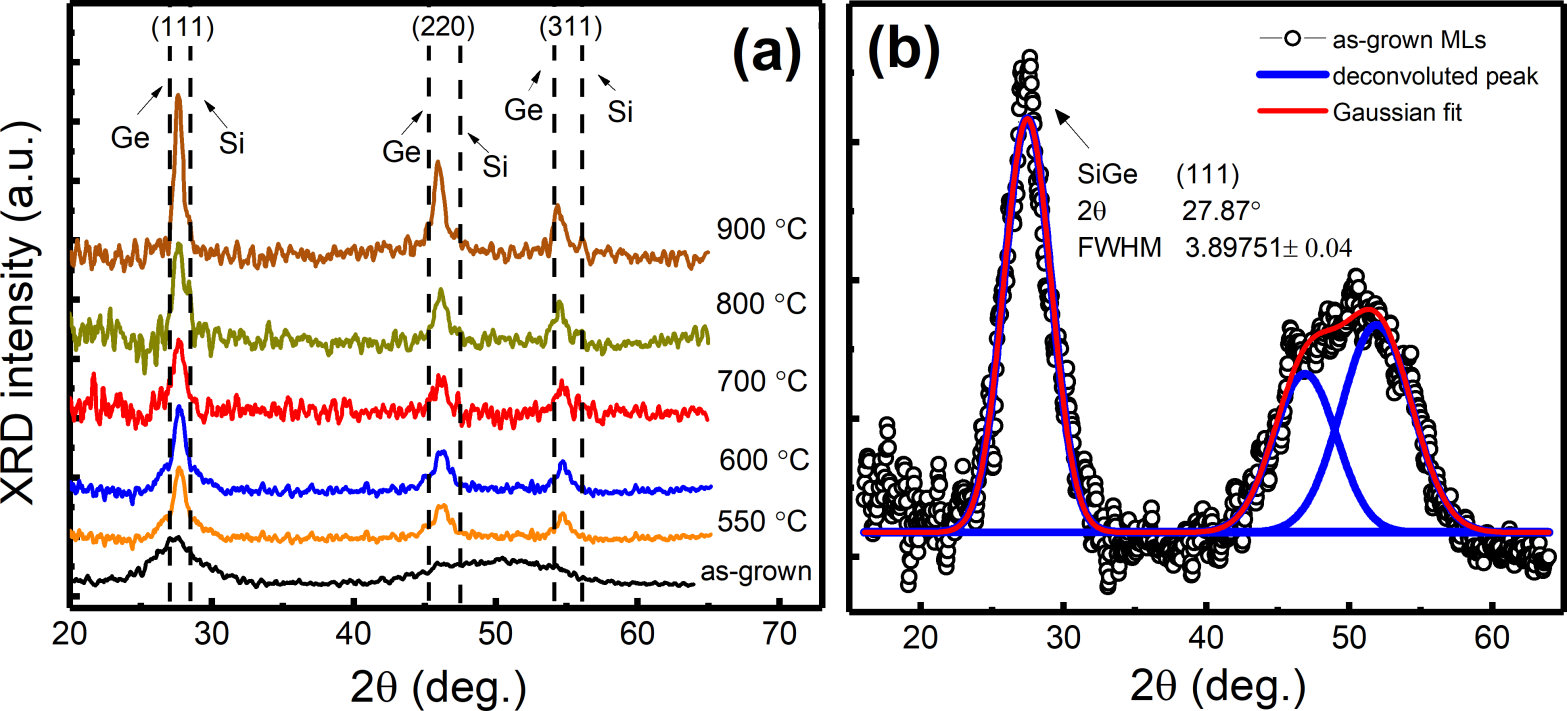
(a) GiXRD diffractograms of MLs annealed from 550–900 °C along with the as-grown MLs. The SiGe crystallographic peaks (111), (220) and (311) are positioned between the tabulated peaks of Si and Ge presented by the dotted lines (for cubic Ge (2θ = 27.45°, 45.59° and 54.04°; ASTM 01-079-0001) and cubic Si (28.45°, 47.31° and 56.13°; ASTM 01-070-5680)). (b) Deconvoluted GIXRD diffractogram for SiO_2_/SiGe/SiO_2_ MLs, as-deposited (black circles) with the Gaussian fits shown by the red line.

Earlier we demonstrated that for structures with a pure Ge-film sandwiched between SiO_2_ layers, the Ge films were crystalline when sputtered by the HiPIMS method due to the high electron density in the plasma (high power density). The higher electron density increases the ionization of Ge sputtered off the target, leading to a better quality of the film through ion bombardment. As described later in the Experimental section and also in our earlier study [22], the Si_1−_*_x_*Ge*_x_* layer was co-deposited via combined dcMS and HiPIMS from Si and Ge targets, respectively. Figure 1a shows the GIXRD diffractograms for the as-grown and annealed MLs (550–900 °C). Two broad reflections are evident for the as-grown structure. The first one corresponds to the (111) planes of SiGe and the second one to the (220) and (311) planes, which overlap indicating the presence of (nano)crystallites [22]. In Figure 1b, a deconvolution of diffractogram for the as-grown MLs was achieved using Origin software (ver. 10.0) (checked using X’Pert HighScore Plus software from PANalytical, ver. 2.2). The size of the crystallites was calculated from the (111) peak using the Scherrer equation [33–34] with a shape factor (*k*) of 0.9 and an instrumental error, i.e., beam broadening of 0.12. Although this is an indecisive approach [22–23], the parameters used to calculate the crystallites size are mentioned in Figure 1b and it was found to be 2.1 ± 0.8 nm. This reduction in crystallite size, compared to previously investigated structures is due to variation in deposition parameters such as cathode voltage.

After annealing, three separate and distinctive peaks are evident (Figure 1a). An increase in the XRD peak intensity was observed along with a decrease in full width at half maximum (FWHM), indicating an increased crystallinity. The size of the NCs was determined, using the (111) peak using the multiple peak feature of Origin (ver. 10.0). It varies from 7.3 to 13.4 ± 0.8 nm in the annealing temperature range from 550 to 900 °C.

**Figure 2:**
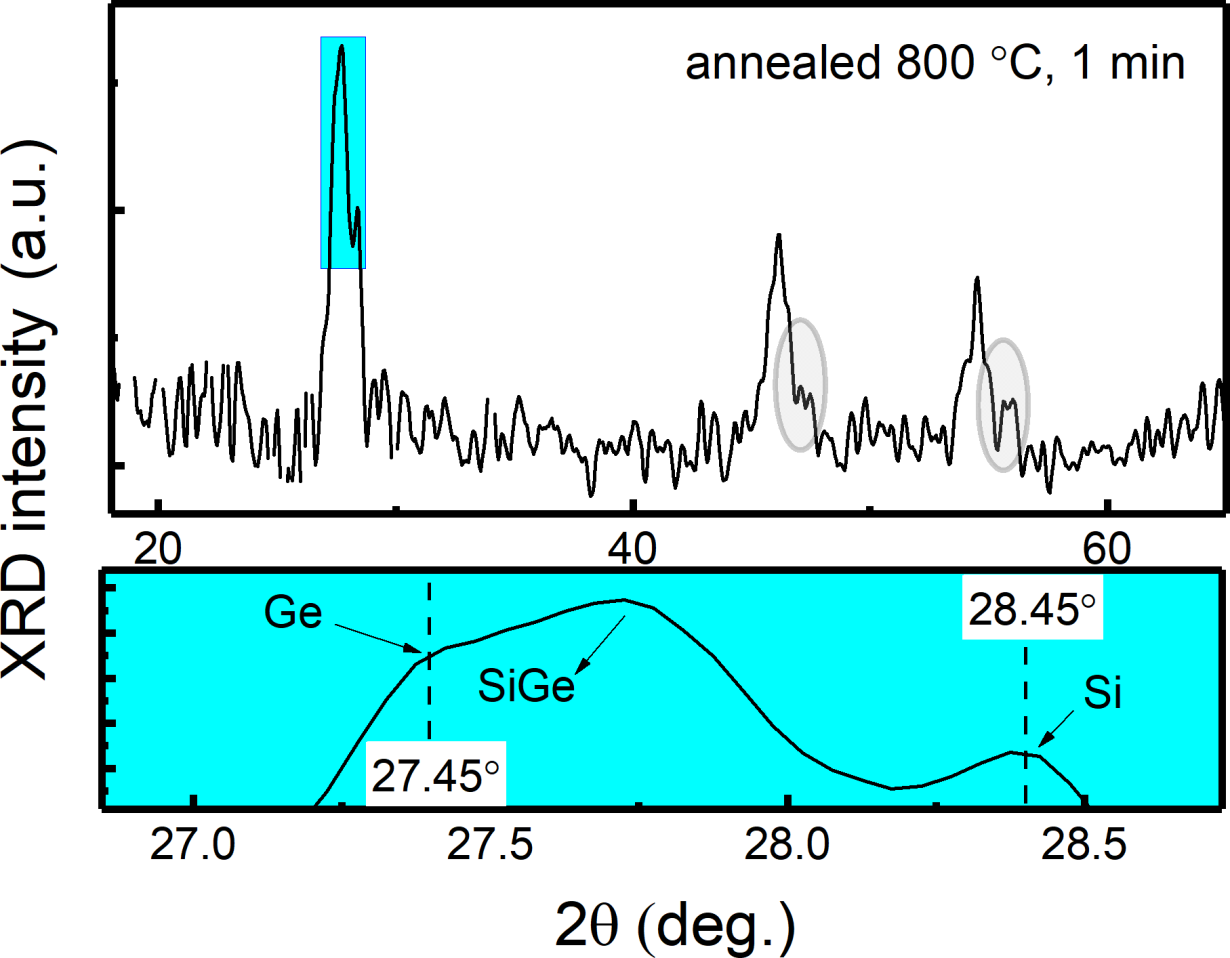
GIXRD diffractogram (upper part) with zoomed-in view (lower part) of crystallographic plane (111) of MLs annealed at 800 °C for 1 min.

Another feature is that, for samples annealed at 550 and 600 °C (Figure 1a), a sharp peak over a broad hump (extending from 25° to 31°) is seen, indicating that the SiGe layer is mainly amorphous but with crystalline regions (nanoparticles) (as seen in TEM images later in Figure 5a and Figure 5c). With increased annealing temperature, peaks corresponding to the (111), (220) and (311) planes get sharper and narrower as a sign of increased crystallinity of the SiGe layer. Moreover, a small peak at a standard Si position (28.45°) is observed at annealing temperatures above 600 °C (Figure 2, selected zoomed view of peak (111) for MLs annealed at 800 °C), along with a shoulder positioned at a standard Ge position (27.45°). Based on these observations, it can be concluded that the structure consists of core–shell NCs/NPs with the core being Ge-rich Si_1−_*_x_*Ge*_x_* NCs (crystallographic peak (111) position, shifts from 27.87° to 27.75° for MLs in as-grown and annealed at 800 °C states, respectively) surrounded by a shell of crystalline Si in amorphous SiGeO. This behavior can be explained by phase separation in the SiGe nanoparticles due to Ge segregation [34–35] at higher temperatures (i.e., Ge-rich SiGe core), which leaves a crystalline Si shell. A similar GIXRD diffractogram was observed by Tuğay et al. [6], for a comparable structure composed of SiGe NCs embedded in a SiO_2_ matrix fabricated via magnetron sputtering and thermal annealing. A TEM analysis discussed below will elaborate on the observed nanostructure.

**Figure 3:**
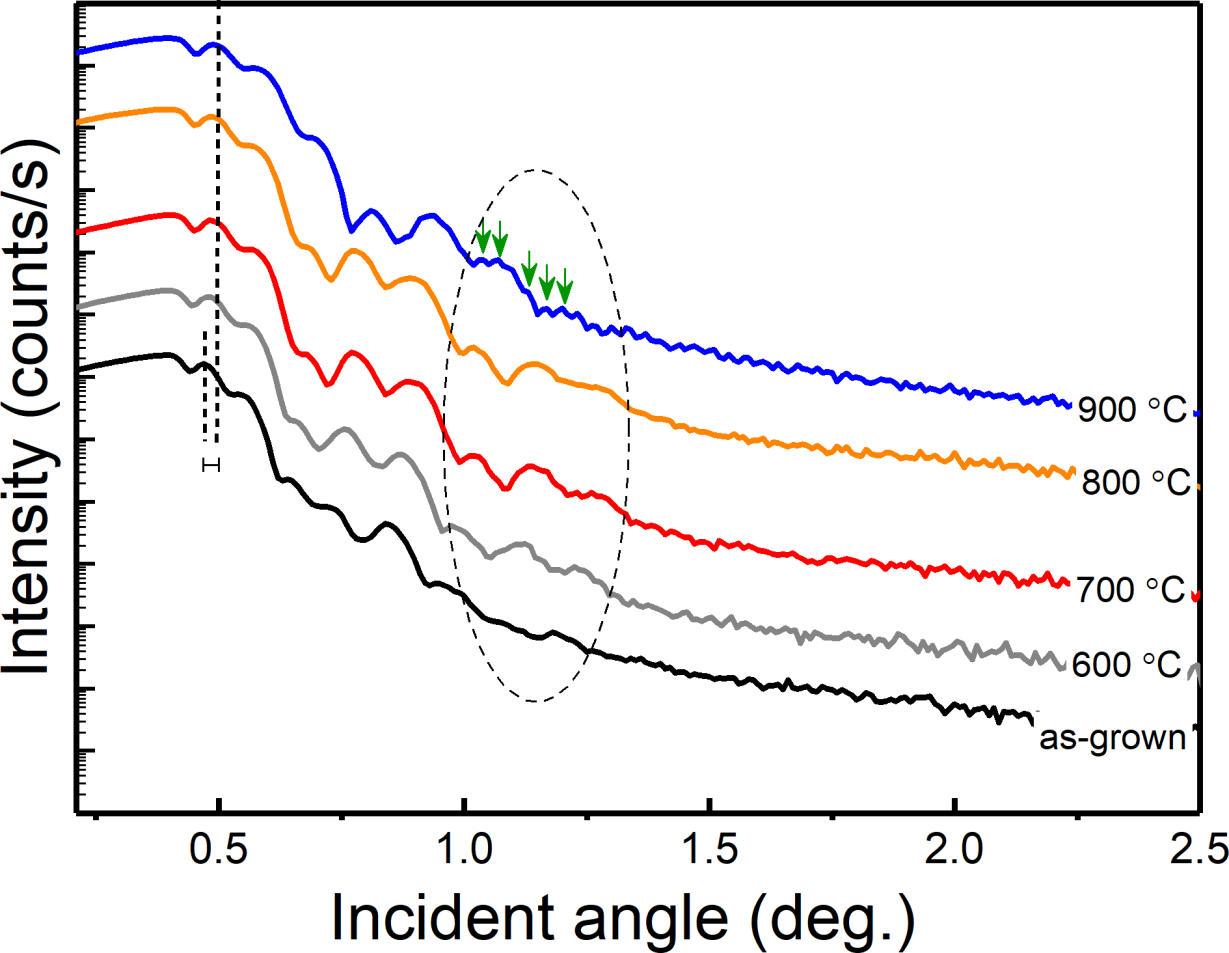
XRR plot for as-deposited and annealed (for 1 min) structures. The vertical dashed lines illustrate the difference in incidence angle.

Figure 3 shows the X-ray reflectometry (XRR) plot for as-deposited and annealed MLs. An increase in the mass density of SiGe (3.55 to 4.17 g/cm^3^) with increased annealing temperature was perceived, represented by the vertical dashed lines. In addition, a decrease in the SiGe thickness (19.57 to 17.8 nm (±3% error)) and the interface roughness (3.56 to 3.28 nm) was observed with increased annealing temperature from room temperature (as-grown) to 900 °C. All parameters were determined by fitting the data using the X’Pert Reflectivity software. A clear evolution of fringes can be seen (shown in an enclosed area by dashed line in Figure 3) for annealing temperatures up to 700 °C. The fringes begin to coalesce at 800 °C and later Kiessig fringes appear (green arrows) due to scattering from the film surface and internal interfaces, thus demonstrating the alteration in the internal interface morphology. This can be further explained by the observed reduction in thickness when annealed at 800–900 °C and might be due to diffusion of Si forming Si shells (as described earlier, when the SiGe(111) peak shifted towards the standard Ge position) or SiO*_x_* (will be discussed later in this section). Hence, with increasing annealing temperatures, the formation of additional interfaces is likely to occur.

**Figure 4:**
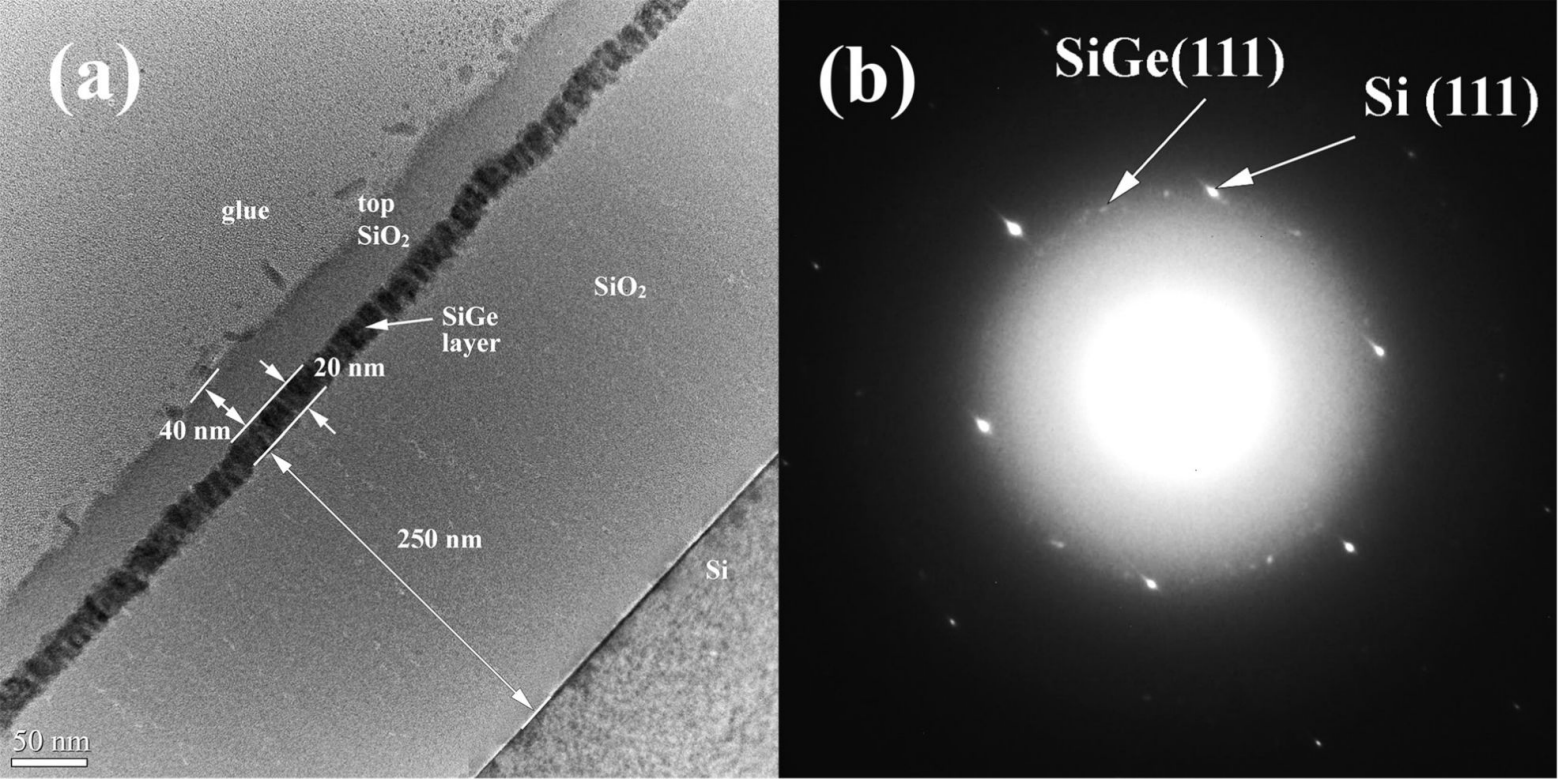
XTEM images of (a) MLs with 20 nm SiGe layer after 600 °C annealing for 1 min, (b) SAED pattern taken on annealed MLs (600 °C, 1 min).

In Figure 4a, a cross-sectional transmission electron microscopy (XTEM) image of the sample annealed at 600 °C for 1 min is presented. The thicknesses of the SiO_2_ bottom (buffer) and top layers are about 250 nm and 40 nm, respectively while the SiGe layer is 20 nm thick. Figure 4b presents the selected area electron diffraction (SAED) pattern. The area used for electron diffraction was selected such that the Si substrate spots together with the ring spots of the SiGe polycrystalline layer were measured. A description of this analysis is given in our previous work [23]. The bright spots are due to Si substrate and the smaller and less bright spots are due to SiGe NCs. The white circular cloud corresponds to amorphous SiO_2_. Our measurements have an estimated error of 0.5% and the results are in good agreement with the XDR measurements, which correspond to 30:70 composition for Si/Ge [36] (i.e., 0.599 nm is the lattice constant measured by XRD calculated using (220) crystallographic plane).

**Figure 5:**
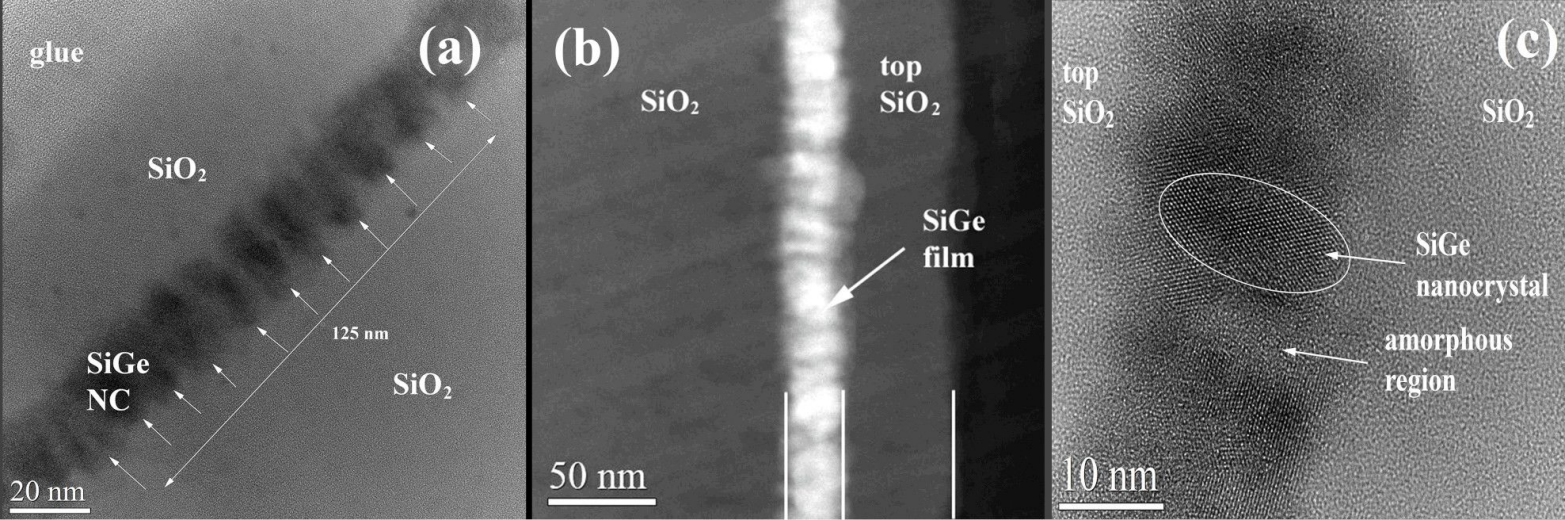
(a) XTEM image of MLs annealed at 600 °C (1 min) showing columnar morphology of SiGe NCs in the film. The crystallites have a periodicity of ≈12.5 nm. (b) STEM-HAADF image. (c) HRTEM image with SiGe NCs separated by amorphous regions (with SiGeO).

The white contrast seen in the high-angle annular dark-field scanning transmission electron microscopy (HAADF-STEM) image (Figure 5b) emphasizes the Ge atoms density, revealing the morphology of the SiGe crystallites. The SiGe NCs have columnar/ellipsoidal morphology oriented with the large axis parallel to the film normal (Figure 5a and Figure 5c). The crystallization process during annealing develops a stress field in the SiGe film plane that is the key factor for obtaining a equidistant/quasiperiodic SiGe NCs arrangement. The SiGe NCs are stress-free in the normal direction on the film and show no internal defects. The formation or modification of the planar morphology of the 20 nm SiGe layer is expected to be due to accumulation of strain exerted by the SiO_2_ matrix, which has been relaxed by forming corrugated edges of the SiGe film (Figure 4a and Figure 5). In contrast, for the thicker SiGe films, the strain is (partially) relaxed by forming planar defects as we demonstrated elsewhere [23] and discuss further below. Periodic SiGe crystallites with a period of ca. 12.5 nm covered with amorphous SiGeO oxide (Si-rich) are visible in Figure 5. The size of 12.5 nm correspond in fact to the diameter of the SiGe ellipsoid plus the thickness of the SiGeO oxide cover-layer, i.e., each SiGe crystallite is covered by 2–3 nm of SiGeO oxide, looking like a core–shell particle. An elemental mapping over a structure (TiO_2_/SiGe/TiO_2_)_3_ annealed at 600 °C in our previous study [37], showed a similar columnar self-assembly of NCs. The analysis showed a well-defined mapping of Si, Ge and Ti (/TiO_2_) with a small fraction of oxygen observed in the SiGe layer. The NCs columns are arranged periodically, having a width of NCs of 10–15 nm, with a gap of 5–6 nm amorphous SiGeO.

We note that the small SiGe nanocrystallites present in as-deposited MLs may have acted as a nuclei for the directional crystallization of the nanoparticles as has also been suggested by Bertan and co-workers [38]. It is postulated there, that the nanosized ordered domains of Si have acted as seed crystals, resulting in a swift growth of crystals upon annealing. A similar phenomenon may have occurred in our structures, as depicted in Figure 4 and Figure 5. Thus, we can anticipate heterogeneous nucleation to be a dominant process during crystallization rather than conventional homogenous nucleation. This can be due to a better wetting of SiGe layer, which in turn reduces the nucleation barrier. It can also be argued that since heterogeneous nucleation occurs at preferential sites (as in our case), small NCs in as-grown MLs or even the crystallites that are under strain [39–42] will further reduce the surface energy and facilitate nucleation.

**Figure 6:**
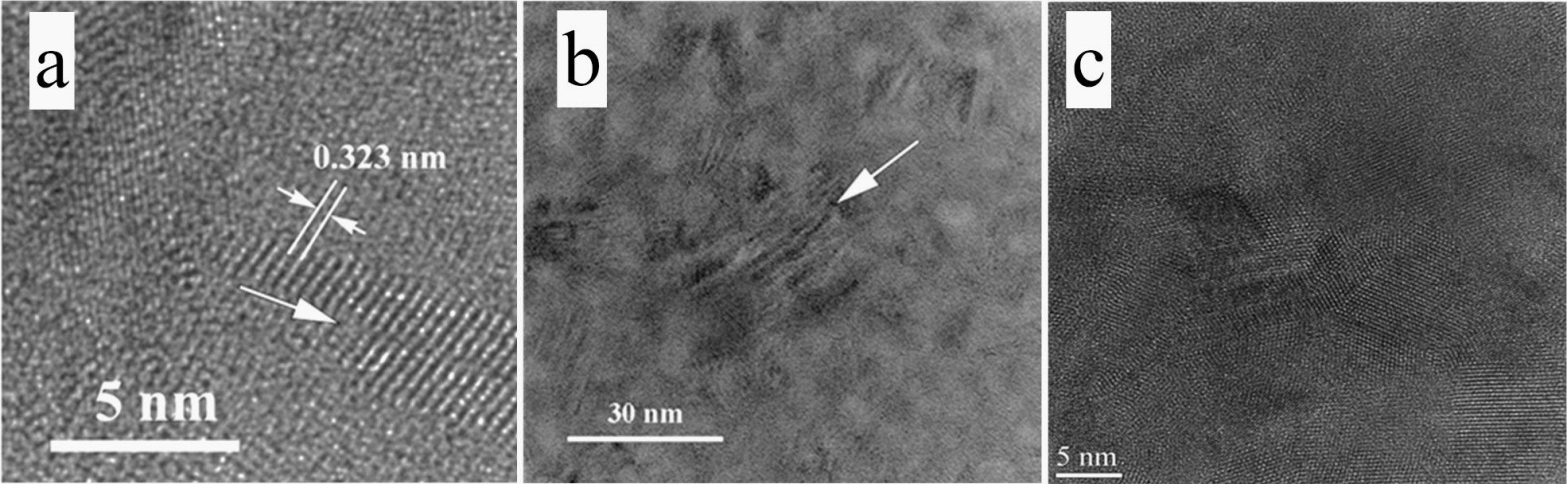
(a) TEM low-magnification image showing the contrast due to the shearing defects appearing in the SiGe crystallites, of a sample annealed at 600 °C for 1 min. (b) High-resolution transmission electron microscope (HRTEM) image showing the lens-like shape of SiGe crystallites as a result of shearing defects. The arrows indicate the shearing planes. (c) Sequence of microtwin bands, observed in a very thin area of the XTEM specimen (MLs with 200 nm thick SiGe [23]). The micrographs in this figure correspond to the structure discussed in our previous study [23].

In order to demonstrate the effect of the SiGe layer thickness on the relaxation processes, Figure 6 depicts micrographs of the previously studied structures [23], where the thickness of the SiGe films was approximately 200 nm. The NCs in the thicker films show a lens-like morphology (Figure 6a), due to the creation of shearing lattice defects (Figure 6b) inside the NCs, which then partially relax the stress field. These planar sharing defects are more complex than the stacking faults and the micro-twins observed [43] in a very thin area of the structure (Figure 6c). In the rest of the specimen area, the shearing defects are superposed and more complicated, as detailed in our previous study [23]. These defects appear only in relatively thick SiGe films in MLs as the only relaxation process taking place. In the thin SiGe films explored here (ca. 20 nm, comparable with the size of the SiGe NCs); these defects do not appear because other relaxation processes take place as shown earlier. Since these shearing defects are near or in the (111) stacking planes of the SiGe structure, the NC size along the direction that is parallel to the defect plane remains large and the two others (related also to the {111} family of planes) are reduced in size, as emphasized in the TEM images in Figure 6. A detailed microstructural TEM analysis of a similar structure has been carried out by Zhang and co-workers [43]. Their analysis revealed that the defects in NCs and twinning in structures is mainly related to the coalescence of small nanoparticles when the structure underwent annealing. A part of the stress in the structure is relieved by the formation of dislocations and the remaining stress is accommodated as local stress at the NC/matrix interface.

### Photocurrent measurements

**Figure 7:**
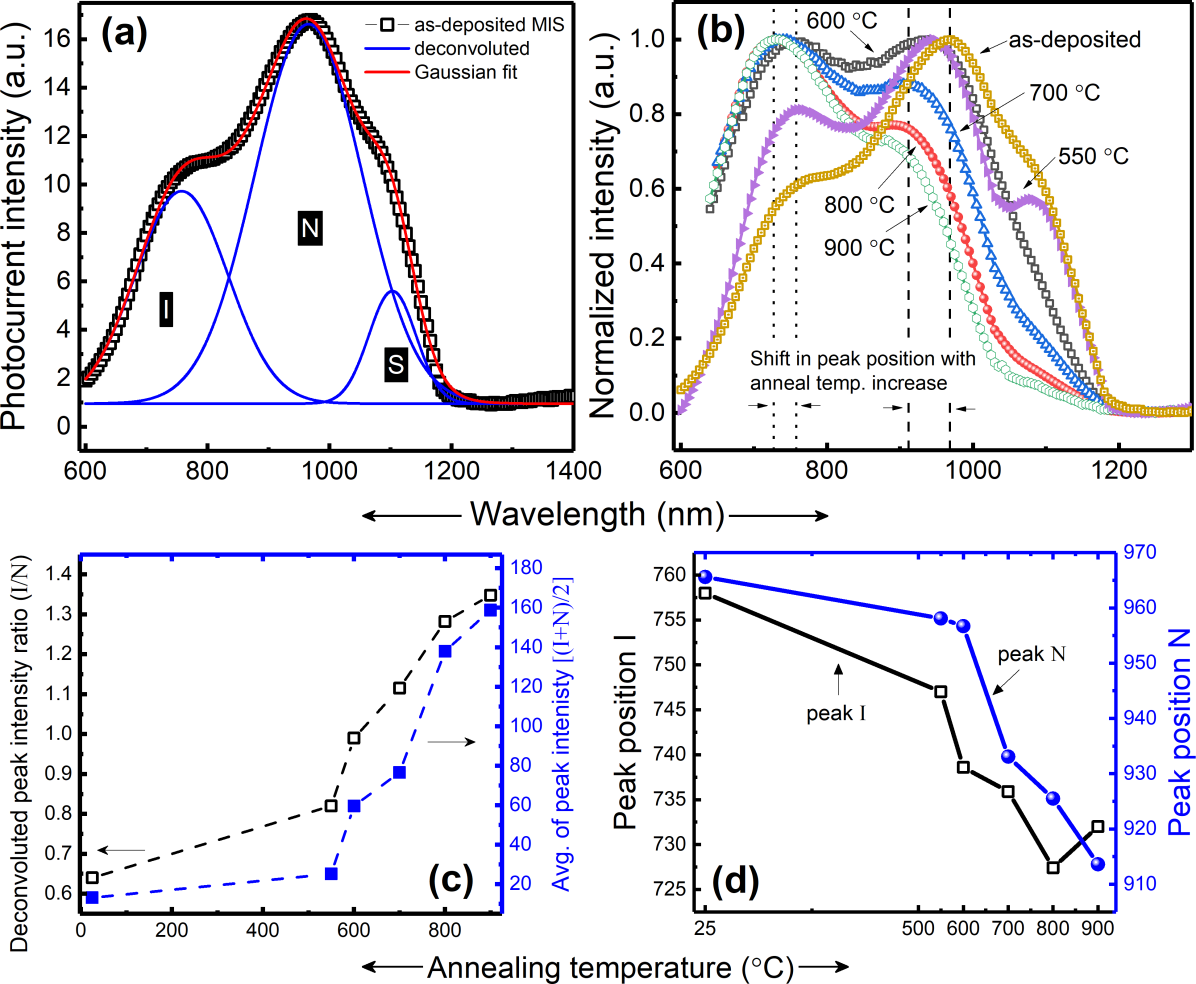
(a) Deconvoluted (Gaussian fit) room-temperature photocurrent spectra of as-grown MLs. (b) Normalized photocurrent spectra of annealed (550–900 °C) and as-grown MLs (the dotted line in the plot represents a blue-shift in peak position with increased annealing temperature). (c) Double *y*-axis plot, with left *y*-axis for alteration in intensity of peak I with respect to peak N (i.e., peak I/N) and right *y*-axis showing an increase in spectral intensity as an average of peaks [(I + N)/2], as a function of the annealing temperature. (d) Peak positions of curves I and N as functions of the temperature for a fixed annealing time of 1 min (values obtained by deconvoluting the spectra).

The photocurrent spectra of as-grown structure (SiGe via dcMS and HiPIMS) are shown in Figure 7a. Deconvolution was carried out to obtain the individual peaks. The observed peaks were assigned to interface related localized states (peak I), the photo effect from NCs (peak N) and capacitive coupling from Si substrate, i.e., surface photo-voltage (SPV) and gating effect (peak S). Figure 7b shows the photocurrent for structures of the same batch that underwent annealing procedure for a short period of 1 min at different temperatures. A large increase in intensity was observed by increased annealing temperature. A more than one order of magnitude higher intensity was obtained upon annealing at 900 °C (Figure 7c, right *y*-axis). In this context, it is worth mentioning that samples with SiGe deposited via dcMS alone resulted in amorphous structure [22], which did not show any measureable photoresponse.

In order to demonstrate clearly the shift in peak positions and the variation in the relative peak intensities, all the spectra Figure 7b were normalized. One can see that with increased annealing temperature, the relative intensity of peak I increases with respect to peak N (also shown in Figure 7c (left *y*-axis) where the ratio of the peak intensities I/N is plotted). This can be explained based on a previous work by Qin and Li [44] who studied interface morphology and related dangling bond affects due to annealing. In light of their results, it has been postulated that there is a critical NCs size above which the interface effect prevails and below which the photoresponse is associated with quantum confinement. That is, size and surface chemistry of the NCs and oxygen-related bonds are the factors determining the photocurrent spectra. It is well understood that annealing results in the formation of dangling bonds in the structures either at the interface of the NCs or in the surrounding matrix [45–47]. Additionally, it is well established, that in the case of increased annealing temperature, a formation of Si=O bonds, along with an increase in the number of dangling bonds may be possible. An increased number of dangling bonds increases the number of localized states in the band structure along with an increase in non-radiative centers (P_b_) [48–49]. This results in a broadening of the energy width of localized states with annealing temperature, resulting in bandgap alteration (Figure 7d shows that both peak I and peak N blue-shift with increasing annealing temperature). These dangling bonds also acts as electrically active recombination centers, which results in an increased photo-response from peak I, hence an increased relative intensity with respect to peak N (Figure 7c).

It has been theoretically shown [50] that Si–O–Si bonds are formed at the surface when a Si NC is oxidized [51]. It is likely that these relatively weak Si–O–Si and Si–Si bonds will break due to stress at the NCs/oxide–matrix interface. Thus, distorted bonds will either result in dangling bonds or eventually form a Si=O bridge, which does not require large additional amounts of energy or deformation to form [52–53]. These dangling bonds, which act as electrically active recombination centers for charge carriers, can alter the optical properties of the structure by contributing to oxide positive charges (depending on the location of the bond) and interface states [45]. One solution to passivate such dangling bonds and/or electrically active recombination centers is the treatment of the structure with hydrogen plasma, as already carried out in our previous study [22] or by annealing the structures in H_2_/N_2_ ambient. This results in the passivation of P_b_-type defects, dangling bonds and oxide fixed charges, increasing the overall sensitivity of the structure. However, the passivation of structures via plasma treatment showed a much better result in terms of increasing spectral sensitivity than that obtained by annealing in H_2_/N_2_ ambient. Additionally, the annealing in H_2_/N_2_ ambient results in a blue-shift of peak N and is limited as H_2_ tends to leave the structure when annealed above 400 °C. To further elucidate the origin of peaks I and N, further studies [22,37] of spectral analysis at varying measurement temperatures (80–300 K) and at varying applied bias (1–11 V) were carried out.

In addition, the annealing of the structure results in reconstruction/ordering of the matrix structure [54], which consequently governs the strain induced on the NCs, and can affect the crystallinity of the NCs [40–41]. The degree of matrix ordering determines the accommodation of the growing crystallites, i.e., the matrix will hinder the NCs to expand freely. Thus, as a result of growing crystallites, strain is introduced at the interface between the matrix and NCs [39–40,55], which in turn alters the bandgap (Figure 7d). Additionally, thermal expansion of SiGe/SiO_2_ and lattice mismatch between Si and Ge (4.2% [31,56]) add to the development of strain in structure and should be taken into account [39]. From the above discussion, it can be summarized that the annealing temperature does affect the structuring of the oxide matrix. This, in turn, induces strain in the structure and therefore alters the interface morphology inducing a change in the intensity ratio between peak I and peak N (Figure 7d).

## Conclusion

SiGe NCs sandwiched between SiO_2_ layers were fabricated by co-sputtering using HiPIMS and dcMS followed by rapid thermal annealing (1 min) at different temperatures. It is shown that HiPIMS deposition facilitates the formation of small nanoparticles/clusters in the as-grown structures. A suitable selection of annealing temperature and time results in the columnar self-assembly of SiGe core–shell NCs, as comprehensively studied by GiXRD and TEM analysis. The self-assembly is attributed to a dominant strain relaxation process, further assisted by already present small nanoparticles in the as-grown structures, acting as seed crystals for heterogeneous nucleation. The photocurrent study reveals that strain and its influence on the NCs/matrix interface morphology play a vital role in determining spectral features and sensitivity.

## Experimental

A multilayer structure with stacking order of SiO_2_/SiGe/SiO_2_ was prepared by magnetron sputtering over a 12 × 12 mm^2^ Si(001) substrate. Prior to deposition, the substrate was etched with 2 M hydrofluoric acid (HF) for 120 s to remove native oxide. For the SiGe films, co-sputtering was carried out from individual targets of (6N purity) Si and Ge. Deposition of Si was carried out via dcMS at 180 W, whereas Ge was sputtered via HiPIMS operating at 445 V cathode voltage at a repetition frequency of 300 Hz. An average power of 103 W, with an average current density and peak power density of 233 mA/cm^2^ and 107 W/cm^2^, was maintained over the full target area. A 3.0″ MAK Planar Magnetron Sputter Source, MeiVac, with Nd/FeB magnets was employed. The Ge target experienced a stronger magnetic field strength |B| than the Si target, opposite to our previous study [22]. Since the deposition rate of Ge is usually higher than that of Si, the |B| is selected accordingly. It has been acknowledged for both dcMS and HiPIMS that the increase in |B| results in a decreased deposition rate (DR) [57–59], and for HiPIMS it often increased the ionized flux fraction. This explanation justifies the need to reconsider the differences in sputter parameters and deposition rates and the resulting change in crystalline size as mentioned in the Results and Discussion section. Additionally, a constant deposition ratio between Si and Ge was maintained in the present study, as confirmed by GiXRD analysis.

For the SiO_2_ layers, deposition was carried out via reactive dcMS sputtering. A detailed description of the sputter technique and equipment used, along with a schematic of as-grown structure is given elsewhere [22]. After deposition, the structure underwent annealing for 1 min in a rapid thermal processor (RTA, Jipelec JetFirst 200) at temperatures ranging from 550 to 900 °C, in N_2_ atmosphere.

The structural investigation of the fabricated MLs was carried out by grazing incidence XRD (GIXRD) and X-ray reflectometry (XRR) via Philips X'pert diffractometer (Cu Kα, 0.15406 nm, precision of 0.00001°) and Jeol ARM 200F transmission electron microscopy (TEM). For the X-ray diffraction scans, a 2×Ge(220) asymmetrical hybrid monochromator utilizing line focus, with a 1/4° divergence slit and a 0.27° parallel plate collimator was used. The measurement run was made over 0.005 °/s scan speed.

For photoconductive measurement, Al contacts (1 × 4 mm^2^) in co-planar geometry with a gap of 4 mm between them were deposited by evaporation. A schematic of the photocurrent setup and the procedure to acquire photo-spectra can be found elsewhere [23].

## References

[1] Tevaarwerk E, Rugheimer P, Castellini O M, Keppel D G, Utley S T, Savage D E, Lagally M G, Eriksson M A (2002). Appl Phys Lett.

[2] Buljan M, Pinto S R C, Kashtiban R J, Rolo A G, Chahboun A, Bangert U, Levichev S, Holý V, Gomes M J M (2009). J Appl Phys.

[3] Mihalache D (2011). J Optoelectron Adv Mater.

[4] Lepadatu A M, Stavarache I, Maraloiu A, Palade C, Serban T V, Magdalena C L (2012). CAS 2012 (International Semiconductor Conference).

[5] Pan S W, Zhou B, Chen S Y, Li C, Huang W, Lai H K (2011). Appl Surf Sci.

[6] Tuğay E, Ilday S, Turan R, Finstad T G (2014). J Lumin.

[7] Vieira E M F, Toudert J, Rolo A G, Parisini A, Leitão J P, Correia M R, Franco N, Alves E, Chahboun A, Martín-Sánchez J (2017). Nanotechnology.

[8] Kepa J, Stesmans A, Afanas’ev V V (2014). Appl Surf Sci.

[9] Houssa M, Pourtois G, Meuris M, Heyns M M, Afanas’ev V V, Stesmans A (2011). Microelectron Eng.

[10] Madia O, Nguyen A P D, Thoan N H, Afanas’ev V, Stesmans A, Souriau L, Slotte J, Tuomisto F (2014). Appl Surf Sci.

[11] Tsetseris L, Pantelides S T (2011). Microelectron Eng.

[12] Lepadatu A-M, Slav A, Palade C, Dascalescu I, Enculescu M, Iftimie S, Lazanu S, Teodorescu V S, Ciurea M L, Stoica T (2018). Sci Rep.

[13] Pinto S R C, Kashtiban R J, Rolo A G, Buljan M, Chahboun A, Bangert U, Barradas N P, Alves E, Gomes M J M (2010). Thin Solid Films.

[14] Stavarache I, Lepadatu A-M, Stoica T, Ciurea M L (2013). Appl Surf Sci.

[15] Choi W K, Kanakaraju S, Shen Z X, Li W S (1999). Appl Surf Sci.

[16] Jie Y X, Wu X, Huan C H A, Wee A T S, Guo Y, Zhang T J, Pan J S, Chai J, Chua S J (1999). Surf Interface Anal.

[17] Vieira E M F, Pinto S R C, Levichev S, Rolo A G, Chahboun A, Buljan M, Barradas N P, Alves E, Bernstorff S, Conde O (2011). Microelectron Eng.

[18] Ray S K, Das S, Singha R K, Manna S, Dhar A (2011). Nanoscale Res Lett.

[19] Chew H G, Choi W K, Foo Y L, Zheng F, Chim W K, Voon Z J, Seow K C, Fitzgerald E A, Lai D M Y (2006). Nanotechnology.

[20] Zschintzsch M, von Borany J, Jeutter N M, Mücklich A (2011). Nanotechnology.

[21] Barradas N P, Alves E, Vieira E M F, Parisini A, Conde O, Martín-Sánchez J, Rolo A G, Chahboun A, Gomes M J M (2014). Nucl Instrum Methods Phys Res, Sect B.

[22] Sultan M T, Gudmundsson J T, Manolescu A, Stoica T, Ciurea M L, Svavarsson H G (2019). Appl Surf Sci.

[23] Sultan M T, Manolescu A, Gudmundsson J T, Torfason K, Alexandru Nemnes G, Stavarache I, Logofatu C, Teodorescu V S, Ciurea M L, Svavarsson H G (2019). Appl Surf Sci.

[24] Palade C, Slav A, Lepadatu A M, Maraloiu A V, Dascalescu I, Iftimie S, Lazanu S, Ciurea M L, Stoica T (2018). Appl Phys Lett.

[25] Helmersson U, Lattemann M, Bohlmark J, Ehiasarian A P, Gudmundsson J T (2006). Thin Solid Films.

[26] Lundin D, Sarakinos K (2012). J Mater Res.

[27] Gudmundsson J T (2010). Vacuum.

[28] Gudmundsson J T, Brenning N, Lundin D, Helmersson U (2012). J Vac Sci Technol, A.

[29] Samuelsson M, Lundin D, Jensen J, Raadu M A, Gudmundsson J T, Helmersson U (2010). Surf Coat Technol.

[30] Alami J, Persson P O Å, Music D, Gudmundsson J T, Bohlmark J, Helmersson U (2005). J Vac Sci Technol, A.

[31] Aqua J-N, Berbezier I, Favre L, Frisch T, Ronda A (2013). Phys Rep.

[32] Stavarache I, Maraloiu V A, Negrila C, Prepelita P, Gruia I, Iordache G (2017). Semicond Sci Technol.

[33] Patterson A L (1939). Phys Rev.

[34] Mogaddam N A P, Alagoz A S, Yerci S, Turan R, Foss S, Finstad T G (2008). J Appl Phys.

[35] Ciurea M L, Lepadatu A M (2015). Dig J Nanomater Bios.

[36] Dismukes J P, Ekstrom L, Paff R J (1964). J Phys Chem.

[37] Sultan M T, Gudmundsson J T, Manolescu A, Teodorescu V S, Ciurea M L, Svavarsson H G (2019). Nanotechnology.

[38] Bertran E, Sharma S N, Viera G, Costa J, St'ahel P, Cabarrocas P R i (1998). J Mater Res.

[39] Bahariqushchi R, Raciti R, Kasapoğlu A E, Gür E, Sezen M, Kalay E, Mirabella S, Aydinli A (2018). Nanotechnology.

[40] Zatryb G, Podhorodecki A, Misiewicz J, Cardin J, Gourbilleau F (2013). Nanoscale Res Lett.

[41] Liao P H, Hsu T C, Chen K H, Cheng T H, Hsu T M, Wang C C, George T, Li P W (2014). Appl Phys Lett.

[42] Zatryb G, Misiewicz J, Wilson P R J, Wojcik J, Mascher P, Podhorodecki A (2014). Thin Solid Films.

[43] Zhang M, Cai R, Zhang Y, Wang C, Wang Y, Ross G G, Barba D (2014). Mater Charact.

[44] Qin G G, Li Y J (2003). Phys Rev B.

[45] Yakimov A I, Kirienko V V, Armbrister V A, Dvurechenskii A V (2014). Semicond Sci Technol.

[46] Brown W D, Khaliq M A (1990). Thin Solid Films.

[47] Dashiell M W, Denker U, Müller C, Costantini G, Manzano C, Kern K, Schmidt O G (2002). Appl Phys Lett.

[48] Nazarov A N, Lysenko V S, Nazarova T M (2008). Semicond Phys, Quantum Electron Optoelectron.

[49] Nikitin T, Khriachtchev L (2015). Nanomaterials.

[50] Wolkin M V, Jorne J, Fauchet P M, Allan G, Delerue C (1999). Phys Rev Lett.

[51] Szekeres A, Alexandrova S (1996). Vacuum.

[52] Puzder A, Williamson A J, Grossman J C, Galli G (2002). J Chem Phys.

[53] López M, Garrido B, García C, Pellegrino P, Pérez-Rodríguez A, Morante J R, Bonafos C, Carrada M, Claverie A (2002). Appl Phys Lett.

[54] Hadjisavvas G, Kelires P C (2004). Phys Rev Lett.

[55] Hadjisavvas G, Remediakis I N, Kelires P C (2006). Phys Rev B.

[56] Ye H, Yu J (2014). Sci Technol Adv Mater.

[57] Ekpe S D, Jimenez F J, Field D J, Davis M J, Dew S K (2009). J Vac Sci Technol, A.

[58] Papa F, Gerdes H, Bandorf R, Ehiasarian A P, Kolev I, Braeuer G, Tietema R, Krug T (2011). Thin Solid Films.

[59] Čapek J, Hála M, Zabeida O, Klemberg-Sapieha J E, Martinu L (2013). J Phys D: Appl Phys.

